# Prophylaxis of Gonorrheal Infection

**Published:** 1901-10

**Authors:** Thomas Marschalko

**Affiliations:** Professor in the University of Klausenburg


### Prophylaxis of Gonorrheal Infection.

BY DR. THOMAS MARSCHALKO,
Professor in the University of Klausenburg.

Although we do not as yet possess a reliable method for preventing syphilitic infections, a fairly certain prophylaxis against gonorrhea has been evolved, and this is the more important since, as is well known, the control of prostitution exerts absolutely no influence upon the prevention and diffusion of gonorrheal affections. It is known positively at the present day that only secretions containing gonococci are capable of producing infection. When such a secretion comes into contact with the mucous membrane which is susceptible to gonorrheal infection, as, for instance, the conjunctiva or urethral mucosa, a certain time always elapses before the gonococci begin to increase. At first, however, the more so as they have no motility, they remain in the same place and can be reached and destroyed by antiseptics. This theory led Crede to propose the prophylactic method with a two per cent. solution of nitrate of silver, known by his name, for the prevention of ophthalmia neonatorum. This procedure has proved most serviceable and may be said to have become a blessing to all mankind, for since Crede's method has been made obligatory, gonorrheal ophthalmia in the new-born, which is the most common cause of blindness during childhood, occurs but scarcely and has lost much of its importance.

The chief requirement of these prophylactic instillations consists in employing the remedy in such quantities and concentration that the gonococci are rapidly and completely destroyed, without causing any marked inflammatory reaction. The next step, therefore, was to test the efficiency of the prophylactic procedure against gonorrheal infection of the male urethra. Gonorrheal infection in the male is due to the fact that in coitus during the relaxation of the erected member after ejaculation some of the gonorrheal secretion from the female genitals is drawn by suction into the meatus. This secretion with its contained gonococci remains adherent for some time to the mucous membrane a few millimeters back of the urethral opening.

A German physician, named Blokusewsky, deserves credit in this direction for taking the initiative. For more than five years he has employed the Crede instillation into the male urethra immediately after, or a short time after, coitus, with the object of preventing gonorrheal infection. These experiments have been completely successful, so that authorities of the first rank, like Neisser, have highly recommended prophylactic instillation for several years.

The two per cent. solution of nitrate of silver, although destructive to the gonococcus in a few seconds, and hence a sure preventive against infection, has the unpleasant effect, even when only one or two drops are instilled as recommended by Blokusewsky, of producing an irritation and causing urethral pains, especially in sensitive persons. This is the reason why Blokusewsky's procedure has not become more popular. When Neisser, however, introduced Protargol into medical practice Dr. Frank, of Berlin, suggested its use as a prophylactic, because this drug, while a powerful gonococcicidal remedy, has the advantages over nitrate of silver of not irritating the urethra even in a ten times stronger concentration, of not being precipitated by albumin or sodium chloride containing solutions, and hence of not being decomposed by the secretions of the body and with it lose its antiseptic action. At the Congress of the German Dermatological Society, in 1898, Dr. Frank referred to a series of experiments made in this direction with a twenty per cent. protargol glycerine solution. This solution was found to destroy the gonococci cultures in test tubes in the course of five seconds as well as in living subjects, and he was able by means of instillations of two or three drops of such a protargol solution to absolutely prevent the development of gonorrheal urethritis. If these experiments are made sufficiently early, and if possible immediately after coitus, I believe that we are perfectly able to prevent gonorrheal infections.

In my own practiceI have tested the prophylactic action of twenty per cent. protargol glycerine instillations with the most satisfactory results, and I would especially cite a case which is most instructive.

Recently a young man consulted me who had relation with one of the demi-monde, from whom he had acquired a severe gonorrhea. As the young man stated that he had had no sexual connection with any one else, I made an examination of his mistress at her own request. This showed that she suffered from a subacute gonorrhea, which, although unattended with any subjective symptoms, still gave rise to a very virulent secretion containing gonococci, as well as from a barthonilitis on the left side. The young man's gonorrhea was completely cured by several weeks' treatment, and during this time he abstained from intercourse. As, however, he was not inclined to give up his illicit connection, I ordered him to use prophylactic instillations of twenty per cent. protargol glycerine solution and urged him never to omit applying them immediately after coitus. Notwithstanding quite frequent intercourse during several weeks he remained unaffected, but on one occasion when he neglected to use the solution after cohabitation, it was at once followed by another gonorrheal infection.

As a matter of fact I order these prophylactic instillations regularly for my patients, and thus far with excellent results.

From what has been said above, I do not hesitate to assert that in the general dissemination of this prophylactic treatment I see the only means of restricting the innumerable cases of gonorrheal infection.

This method has, indeed, been warmly recommended by prominent authorities in all the works on prophylaxis of venereal diseases that have recently appeared. I would, therefore, consider it the duty of every humanitarian physician to further its use among the laity. Not only the specialist, but every practicing physician should know that the most reliable safeguard against gonorrheal infection consists in the use of these instillations immediately after coitus, as they can be easily carried out by any one without the least unpleasant after effects.

Various apparatus have been suggested for this purpose which still further facilitate the employment of this method.—*Die Heilkunde,* V, Vol. 6, June, 1901.

